# MagSculptor: A Microfluidic Platform for High-Resolution Magnetic Fractionation of Low-Expression Cell Subtypes

**DOI:** 10.3390/bios16010041

**Published:** 2026-01-04

**Authors:** Zhenwei Liang, Yujiao Wang, Xuanhe Zhang, Yiqing Chen, Guoxu Yu, Xiaolei Guo, Yuan Ma, Jiadao Wang

**Affiliations:** 1 Department of Mechanical Engineering, Tsinghua University, Beijing 100084, China; 2 State Key Laboratory of Tribology, Tsinghua University, Beijing 100084, China; 3 Center for Medical Device Evaluation, National Medical Products Administration, Beijing 100081, China

**Keywords:** magnetic field shaping, microfluidic cell sorting, immunomagnetic separation, low-expression cell subtypes, EpCAM, whole blood

## Abstract

Heterogeneous expression of a single surface protein within one cell population can drive major functional differences, yet low-expression subtypes remain difficult to isolate. Conventional tube-based immunomagnetic separation collapses all labelled cells into one positive fraction and thus cannot resolve small differences in marker abundance. Here, we present MagSculptor, a microfluidic platform for high-resolution magnetic fractionation of low-expression EpCAM-defined subtypes within one immunomagnetically labelled population at a time. Arrays of soft-magnetic strips create localized high-gradient zones that map modest differences in bead loading onto distinct capture positions, yielding High (H), Medium (M), Low (L), and Negative (N) fractions. Finite element method simulations of coupled magnetic and hydrodynamic fields quantify the field gradients and define an operating window. Experimentally, epithelial cancer cell lines processed sequentially under identical settings show reproducible subtype partitioning. In a low-EpCAM model (MDA-MB-231), conventional flow cytometry, under standard EpCAM staining conditions, did not yield a robust EpCAM-positive gate, whereas MagSculptor still revealed graded subpopulations. Western blotting confirms a monotonic decrease in EpCAM abundance from H to N, and doxorubicin assays show distinct in vitro drug sensitivities, while viability remains above 95%. MagSculptor thus helps extend immunomagnetic separation from binary enrichment to multi-level isolation of low-expression subtypes and provides a convenient front-end for downstream functional and molecular analyses.

## 1. Introduction

Heterogeneity in the expression of cell surface proteins is one of the key regulatory factors governing cellular functions such as differentiation, migration, signal transduction, and immune response [1,2]. In immunotherapy, tumor microenvironment analysis, and early disease diagnostics, the ability to identify and isolate subpopulations with low biomarker expression has both scientific and clinical importance [3]. For example, subtype-specific expression of Cluster of Differentiation (CD) molecules on CD4^+^/CD8^+^ T cells directly influences antigen recognition sensitivity and the duration of immune activity, and is a critical determinant of CAR-T therapeutic efficacy and functional heterogeneity [4,5]. Similarly, epithelial cell adhesion molecule (EpCAM) expression in solid tumors, including breast cancer, correlates with metastatic potential and stem-like properties, and has therefore become a widely used target in liquid-biopsy technologies [6,7,8,9,10,11]. However, when transcriptional expression of a target marker falls below 100 nTPM, conventional cell sorting methods often lack the sensitivity and resolution needed to distinguish subtypes based on expression level rather than merely on marker presence or absence [12].

Magnetic-activated cell sorting (MACS) has become a mainstay for cell isolation owing to its specificity and scalability [13,14]. In typical tube-based workflows, magnetic microbeads are conjugated to target cells via antibody-mediated recognition and captured under an applied field. Because these systems rely on static, spatially homogeneous magnetic field gradients, the available magnetic-force resolution is limited. Cells that differ by only a few percent in magnetic labelling experience effectively indistinguishable capture forces and are therefore collected into a single labelled fraction [15,16,17,18]. As a result, MACS provides reliable positive/negative enrichment but cannot resolve intra-line heterogeneity, such as subtle EpCAM-defined subtypes or graded CAR expression within a T-cell product, which are increasingly recognized as biologically and clinically relevant [19].

To move beyond binary enrichment, several microfluidic platforms have introduced spatially structured magnetic forces. Concepts from high-gradient ferromagnetic particle separation in tribology have been adapted to generate position-dependent field gradients that segregate metallic particles by size or magnetization [20]. For biological applications, intrinsically magnetic cells or bead-labelled cells have been sorted using micro-magnet arrays, visual feedback, and flow-control units to create local capture traps or multi-stage migration paths [21,22,23]. Kelley and co-workers, for example, used arrays of micromagnets and flow-control modules to achieve gradient capture of lymphocyte subtypes [24], while Di Carlo and co-workers implemented multi-directional magnetic ratcheting to encode differences in bead number into differences in travel distance [25]. Ferrohydrodynamic designs have further demonstrated multi-group separation of rare cells through quantitatively engineered flow and magnetic fields [26]. These studies collectively show that magnetic-force differentials can in principle be harnessed for subtype discrimination, but they also highlight trade-offs among force resolution, device complexity, fraction retrieval, and throughput. These trade-offs become particularly restrictive when the magnetic contrast between subtypes is intrinsically small under low-expression conditions.

In this work, we present MagSculptor, a microfluidic platform designed to address these challenges by combining soft-magnetic field shaping with a simple, elution-compatible channel architecture. Arrays of annealed amorphous 1J85 nickel–iron soft-magnetic strips are integrated beneath a straight polycarbonate microchannel and energized by an externally adjustable lateral magnetic field [16,27]. The high permeability of the soft-magnetic material sculpts a series of localized high-gradient zones along the flow path, creating a magnetic landscape in which bead-labelled cells experience position-dependent forces. During each batch run, an EpCAM-labelled cell population is driven through this landscape under well-defined flow conditions, and modest differences in magnetic bead loading are converted into distinct capture positions on the strip array. This enables quantitative mechanical fractionation of a single immunomagnetically labelled population into High (H), Medium (M), Low (L), and Negative (N) subgroups rather than simultaneous separation of different cell lines. We use epithelial cancer cell lines with varying EpCAM expression levels as a model system and sequentially process different lines in separate runs under identical conditions to demonstrate generality. In the following sections, we describe the physical principles, microfluidic design, and finite element method (FEM) simulations of coupled magnetic and hydrodynamic fields that inform device parameters, and we experimentally validate that MagSculptor can resolve low-expression EpCAM subtypes and support downstream functional and molecular analyses. In practice, MagSculptor can operate in two complementary modes: (i) as an immunomagnetic front-end that retrieves the cohort carrying a chosen surface marker from a complex matrix such as whole blood, and (ii) as a high-resolution fractionator that, once such a cohort is obtained, resolves it into expression-based subtypes (H/M/L/N), including cohorts with very low antigen expression.

## 2. Materials and Methods

### 2.1. Cell Culture

The MDA-MB-231, MCF-7, A549, and Caco-2 cell lines were purchased from the National Infrastructure of Cell Line Resource (Beijing, China). MDA-MB-231 and A549 cells were cultured in RPMI-1640 medium supplemented with 10% fetal bovine serum (FBS; Thermo Fisher Scientific, Waltham, MA, USA) and 1% penicillin-streptomycin (Thermo Fisher Scientific, Waltham, MA, USA). MCF-7 and Caco-2 cells were cultured in DMEM high-glucose medium with 10% FBS and 1% penicillin-streptomycin. All cells were maintained in a humidified incubator at 37 °C with 5% CO_2_ and 95% humidity.

### 2.2. Immunomagnetic Labeling

The MDA-MB-231, MCF-7, A549, and Caco-2 cells in good condition were collected and incubated with Calcein-AM (HY-D0041; MedChemExpress, Monmouth Junction, NJ, USA) at 37 °C for 10 min. After incubation, the cells were washed three times with phosphate-buffered saline (PBS) and resuspended in PBS to a concentration of 2 × 10^5^ cells/mL. For immunomagnetic labelling, EpCAM-positive cells were targeted using anti-EpCAM-coated magnetic beads (Exosome-Human EpCAM Isolation Reagent, Dynabeads^TM^, 10618D; Thermo Fisher Scientific, Waltham, MA, USA; 2.7–2.8 µm diameter). Unless otherwise specified, beads were added at a dosage of 75 µL of bead suspension per 1 × 10^6^ cells (corresponding to 15 µL per mL at 2 × 10^5^ cells/mL), following the manufacturer’s recommendations. The bead–cell suspension was incubated at room temperature for 30 min on a gentle rotator to promote binding. The resulting mixture, containing both bead-labelled cells and a residual population of unbound beads, was then directly introduced into the MagSculptor device without additional washing. In our experiments, free beads were rapidly scavenged by the upstream region of the strip array and accumulated predominantly near the inlet, and did not appreciably contaminate the downstream capture positions used for subtype analysis. The same labelling protocol was used for all four cell lines unless otherwise specified. For whole-blood spike-in experiments, the Calcein-AM fluorescence label on the spiked tumor cells was retained so that these cells could be distinguished from endogenous blood cells during image-based quantification of capture efficiencies.

### 2.3. Fabrication of Soft Magnetic Array

For magnetic field shaping, we used annealed amorphous 1J85 nickel–iron soft-magnetic alloy as the strip material. A 35 µm-thick 1J85 sheet was bonded to glass substrates using epoxy resin. A positive photoresist (S1813) was spin-coated uniformly, soft-baked, exposed through a chrome mask defining the strip pattern, and developed to generate the etch mask. The exposed 1J85 was etched using a peroxide-enhanced hydrochloric acid solution delivered as a uniform spray until the underlying glass was reached. After etching, the remaining photoresist protected the strip regions. Using this lithography–etch process, the soft-magnetic strip array exhibited a within-lot strip-width deviation within ±10% of the nominal design, which was sufficient to reproducibly shape the graded magnetic field required by MagSculptor. Representative photographs and microscope images of the etched arrays are shown in Figure S1. Additional geometric tolerances and fabrication details for the strip array are provided in Supplementary Method S-M2.

### 2.4. Assembling of MagSculptor

The MagSculptor device consisted of three core components: (i) a glass substrate patterned with the soft-magnetic strip array, (ii) a thermoplastic polycarbonate (PC) microfluidic layer, and (iii) top and bottom acrylic clamping plates. The soft-magnetic array comprised 35 µm thick parallel strips with a designed width and pitch of 500 µm. During process optimization, narrower 100 µm strips could be fabricated but they generated insufficient magnetic capture forces under the operating conditions; therefore, a 500 µm linewidth configuration was selected as a compromise between capture strength and inter-group differentiation. Each strip extended 6 mm in the transverse (y) direction, spanning y = −3 to 3 mm and fully covering the 4 mm-wide microchannel to ensure complete magnetic field coverage across the flow path.

The microchannel was constructed as a three-layer PC laminate: a top port plate, a 100 µm laser-cut middle spacer that defined the channel geometry and height, and an intact 50 µm bottom PC film that provided continuous electrical and chemical insulation from the soft-magnetic array. The laminate was aligned and clamped over the strip substrate with the 50 µm PC film interposed, thereby setting the strip–channel gap (D = 50 µm) and preventing direct contact between the fluid and the metal. This configuration prevented saline-induced corrosion while preserving the designed magnetic field across the gap. The soft-magnetic strips remained air-exposed outside the sealed channel.

The assembled chip was sandwiched between two 3-mm-thick CNC-milled polymethyl methacrylate (PMMA) plates, and uniformly compressed using screws and nuts to ensure a tight, leak-free seal. Inlets and outlets were connected via standard Luer-lock fittings to external syringe pumps for controlled fluid delivery. A more detailed description of the device stack, bonding and clamping strategy, and surface-conditioning protocol is provided in Supplementary Method S-M2. A neodymium-core electromagnet was positioned laterally, with its central axis aligned coplanar to the strip array, to provide an adjustable background magnetic field.

### 2.5. Theoretical Model

Finite element method (FEM) simulations were carried out in COMSOL Multiphysics^®^ (version 5.6, COMSOL AB, Stockholm, Sweden) using the Magnetic Fields interface (AC/DC Module) coupled to Laminar Flow (incompressible) for the microchannel; where the sample/buffer interface was illustrated, we used Two-Phase Flow, Phase Field. Additional details of the FEM geometry, material properties, boundary conditions, mesh refinement strategy, and particle-trajectory computations are provided in Supplementary Method S-M1. The only custom term is the magnetophoretic volume force applied to bead-labelled cells, which can be expressed as Equation (1) [28]:

(1) $$F_{m}=n_{b}\mu_{0}V_{b}\frac{3(\chi_{b}-\chi_{f})}{(\chi_{b}-\chi_{f})+3}(H\cdot \nabla )H$$

where *μ*_0_ = 4π × 10^−7^ T·m/A represents the permeability of free space, *n_b_* denotes the number of magnetic beads bound to a single cell, and *χ_c_* and *χ_f_* represent the susceptibility of the beads and the fluid environment, respectively. Any magnetically labelled cell experiences forces within the magnetic field that are directly proportional to both the intrinsic properties of the cell-bead conjugate and the magnetic field characteristics are mathematically expressed as (***H***·∇)***H***. 

We modeled the magnetic force from the standard susceptibility contrast form **F**_m_∝(***H***·∇)***H*** and inserted it into the momentum equation. All other governing equations and couplings follow the default module formulations. Boundary conditions included magnetic insulation/continuity at material interfaces and no-slip walls with fully developed laminar inflow and pressure outlet; mesh and solver settings followed COMSOL defaults with parametric sweeps over strip geometry and gap D.

### 2.6. Operating Parameters of MagSculptor

Prior to each experimental session, the chip was conditioned by flushing with 1% Pluronic F-127 to minimize nonspecific adhesion and ensure reproducibility. Samples were prepared with 2 × 10^5^ cells/mL and incubated with magnetic beads under optimized binding conditions. During subtype-gradient separation in PBS-based experiments, the sample was introduced at a flow rate of 3000 µL/h while 1× PBS buffer was co-infused at a flow rate of 1000 µL/h to stabilize the phase interface and maintain separation, corresponding to a 3:1 sample-to-buffer ratio and a total flow rate of 4000 µL/h. Given the 4 mm × 100 µm channel cross-section and the 27 mm length of the strip array, these conditions yield an average axial velocity of ≈2.8 mm/s and an estimated residence time of ≈10 s for cells traversing the sorting zone. At a concentration of 2 × 10^5^ cells/mL, this flow configuration corresponds to a nominal cell throughput of ≈8 × 10^5^ cells/h when the device is operated continuously.

To remove any non-magnetic residuals after sample loading, the original flow rates were maintained while an additional ~200 µL of PBS was flushed through the channel. Each flow channel could complete the process independently, and the modular parallel-channel architecture enabled higher throughput (see Figure S2a for the workflow). Additional details are provided in Supplementary Method S-M1.

Effluent was collected at the outlet under ambient pressure to ensure gentle handling of cells. During fraction collection, the total flow rate was gradually increased in a controlled manner so that bead-labelled cells were sequentially released and collected into distinct subgroups according to their magnetic bead content. The off-chip setup is illustrated in Figure S2b; a suspended pipette-tip reservoir provided bubble-free loading and allowed for fine adjustment of hydrostatic head.

For comparative experiments across different cell lines, samples were loaded one at a time into the same device while keeping the magnetic field strength, flow rate, cell concentration, and bead-incubation conditions constant. After each capture and imaging cycle, the background magnetic field was turned off, and the channel was rinsed with PBS at a flow rate of 60,000 μL/h to release previously captured cells and prevent carryover before the next run. Panels are pseudo-colored in post-processing for visual distinction of the four separate sequential experiments; all images were acquired using the same fluorescence channel. Pseudo-coloring does not indicate simultaneous co-loading of different cell lines.

### 2.7. Cell Survival Rate Detection Method

Following subtype sorting (Section 2.6), each fraction (H, M, L, and N) was resuspended to a concentration of 2 × 10^5^ cells/mL and stained with Calcein-AM and propidium iodide (PI) at 37 °C for 10 min. For each fraction, fluorescence images were acquired from at least three non-overlapping fields of view per experiment, and all cells within those fields were counted (typically >200 cells per fraction per replicate). Cells that were Calcein-positive and PI-negative were scored as viable, whereas PI-positive cells (with or without Calcein signal) were scored as non-viable. Thresholds for the green and red fluorescence channels were kept fixed across all images, and counts were performed manually using ImageJ (version 1.51, National Institutes of Health, Bethesda, MD, USA). The viability of each subgroup (H, M, L, and N) was expressed as the percentage of viable cells relative to the total number of cells analyzed in that subgroup.

### 2.8. Drug-Response Assay for Sorted MCF-7 Subtypes

MCF-7 cells were sorted into high (H), medium (M), and low (L) subgroups using the MagSculptor and seeded in 96-well plates at a density of 5000 cells per well. All drug-response assays were performed off-chip on these sorted cells; no additional microfluidic flow through the MagSculptor device was applied during drug exposure. After overnight attachment, the cells were treated with various concentrations of doxorubicin (0, 0.375, 0.625, 1.25, 2.5, 5, and 10 μM) for 48 h, with three technical replicates for each concentration and subgroup. Cell viability was quantified using a CCK-8 assay kit (MedChemExpress, Monmouth Junction, NJ, USA), and the half-maximal inhibitory concentration (IC_50_) was derived from dose–response curves generated using GraphPad Prism (version 9.5, GraphPad Software, San Diego, CA, USA).

### 2.9. Detection of EpCAM Protein Expression Across Different Subgroups by Western Blot

MCF-7 cells were sorted into high (H), medium (M), low (L), and negative (N) subgroups using the MagSculptor system and then lysed using RIPA buffer supplemented with a protease and phosphatase inhibitor cocktail. The protein concentration was determined with a BCA assay kit (Thermo Fisher Scientific, Waltham, MA, USA). Subsequently, proteins (30 µg per lane) were separated by SDS-PAGE and electrophoretically transferred onto PVDF membranes. The membranes were blocked with 5% non-fat milk in TBST for 1 h at room temperature and incubated with primary antibodies against EpCAM and β-actin overnight at 4 °C. After thorough washing, the membranes were incubated with an HRP-conjugated secondary antibody for 1 h at room temperature. The protein bands were visualized by enhanced chemiluminescence (ECL) and imaged using a chemiluminescence system. β-Actin was used as a loading control for normalization.

### 2.10. Flow Cytometry Test

The MDA-MB-231, MCF-7, A549, and Caco-2 cells were collected and resuspended in PBS at a concentration of 1 × 10^6^ cells/100 µL. Then, 2 µL of EpCAM-FITC antibody (MA1-10197, Thermo Fisher Scientific, Waltham, MA, USA) was added, and the mixture was incubated in the dark with gentle rotation for 30 min. Flow cytometry was subsequently used to assess the proportion of EpCAM-negative populations in MDA-MB-231, MCF-7, A549 and Caco-2 cells.

## 3. Results and Discussion

### 3.1. Magnetic Field Shaping

**Figure 1:**
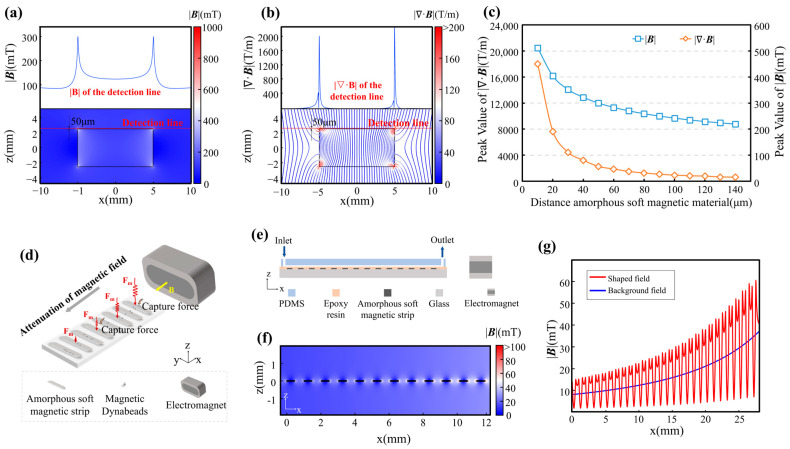
Structural and magnetic field characteristics of the MagSculptor system. (**a**) Magnetic field enhancement near soft magnetic materials. The upper panel illustrates the magnetic field distribution along the detection line depicted in the lower panel. (**b**) Magnetic flux lines and corresponding gradient fields show strong convergence near strip edges. The upper panel delineates the magnetic field gradient along the detection line presented in the lower panel. The color of the flux lines encodes the local magnitude of the magnetic field gradient. (**c**) Magnetic flux density and gradient decay as a function of increasing distance from the soft magnetic element. (**d**) On-axis schematic of the chip with a lateral external field B applied along +x by a side magnet/electromagnet (N/S indicated). Flow is along +x and the soft-magnetic strips are oriented perpendicular to the flow (schematic not to scale; magnet body enlarged for clarity). (**e**) Cross-sectional sketch of the microfluidic chip; schematic not to scale. (**f**) Device-scale simulated spatial distribution of the magnetic field within the channel (actual strip-array geometry and channel dimensions). (**g**) Magnetic field profile at a height of 50 μm above the strip array.

When a high-permeability soft-magnetic element is placed in an external field, it becomes magnetized and redistributes the surrounding field. To separate the field-shaping principle from device-specific details, we first simulated a simplified soft-magnetic block (Figure 1a,b) using the same material properties and magnet–strip spacing as in the microfluidic device. The simulations show pronounced flux concentration at the block edges and large local field gradients, with gradient magnitudes exceeding ~2000 T·m^−1^ at distances on the order of 50 μm from the boundary. These values correspond to the annealed amorphous 1J85 nickel–iron alloy used here (initial permeability ~8 × 10^4^, maximum ~2 × 10^5^). We refer to this edge-enhanced, geometry-dependent redistribution of the field as magnetic field sculpting.

As with conventional permanent magnets, the influence of the soft-magnetic strips decays rapidly with distance. Both the magnetic flux density |B| and the magnitude of its spatial gradient decrease as one moves away from the strip surface (Figure 1c). In our geometry, the gradient of |B| can reach ~1.8 × 10^4^ T·m^−1^ at 10 μm above the strip but falls to ~2 × 10^3^ T·m^−1^ at 50 μm. We therefore chose a 50 μm strip–channel spacing as a compromise between manufacturability, mechanical integrity of bonding, and sufficient magnetic force for capture. Under these conditions, the induced magnetic moment of each bead–cell conjugate experiences a translational force jointly determined by the local field strength and gradient. The requirement to sustain high gradients across the 50 μm-thick flow channel thus becomes a primary design constraint that shapes the overall MagSculptor architecture.

### 3.2. Microfluidic Design and Magnetic Field Coupling

In conventional magnetic cell sorting workflows, target cells are rendered magnetically responsive by antibody-mediated attachment of superparamagnetic beads, and the bead load correlates with the expression level of the surface marker [29]. For low-expression subtypes, the resulting magnetization is weak, so modest differences in bead number translate into only small differences in magnetic force. To resolve such closely spaced subpopulations, the microchannel must provide strong, spatially localized gradients that amplify these differences and convert them into distinct capture positions.

To achieve this, we patterned an array of parallel soft-magnetic strips that are magnetized by a side-mounted, tunable electromagnet (Figure 1d). The slowly varying distance between each strip and the external magnet sets a background field along the channel, while the strips themselves locally concentrate flux and create force wells (Figure 1e). When combined with the laminar flow, this architecture acts as a graded series of capture barriers: cells with higher bead loads are retained on upstream strips, whereas weakly labelled or unlabeled cells travel further downstream.

We describe the geometry in a Cartesian coordinate system with the main flow direction along x. Twenty-seven strips span x ∈ [0, 27] mm, and the face of the electromagnet is located at x = 45 mm; the strip closest to the magnet therefore lies at x = 27 mm (=18 mm from the magnet surface). To avoid confusion, we index strips by position rather than by “strength”: cells enter near x ≈ 0 mm (weakest magnetization) and traverse toward x = 27 mm (strongest magnetization), which is encountered last. This monotonic increase in field strength yields a progressive capture landscape in which magnetic content is translated into capture position. Vertically, the central axis of the electromagnet is aligned with the plane of the strip array, and FEM simulations show that the amplification effect of the strips is maximal within ~50 μm above the strip surface—coinciding with the mid-plane of the 100 μm-tall microchannel (Figure 1f). In this region, narrow peaks in flux density appear near each strip edge, forming high-gradient capture zones.

FEM simulations under the operational field strengths (Figure 1g) show that the background magnetic field, which would otherwise decay gradually from ~35 mT to ~9 mT between x = 27 mm and x = 0 mm, is reshaped by the strip array into an oscillatory profile with a series of local maxima and associated high-gradient bands at the strip boundaries. These simulated hotspots define the principal capture sites. Because the magnetic adsorption force on each bead–cell conjugate depends on both the local field magnitude and its gradient, positioning these hotspots within the flow cross-section is central to the design of MagSculptor and motivates the term “field sculpting”.

**Figure 2:**
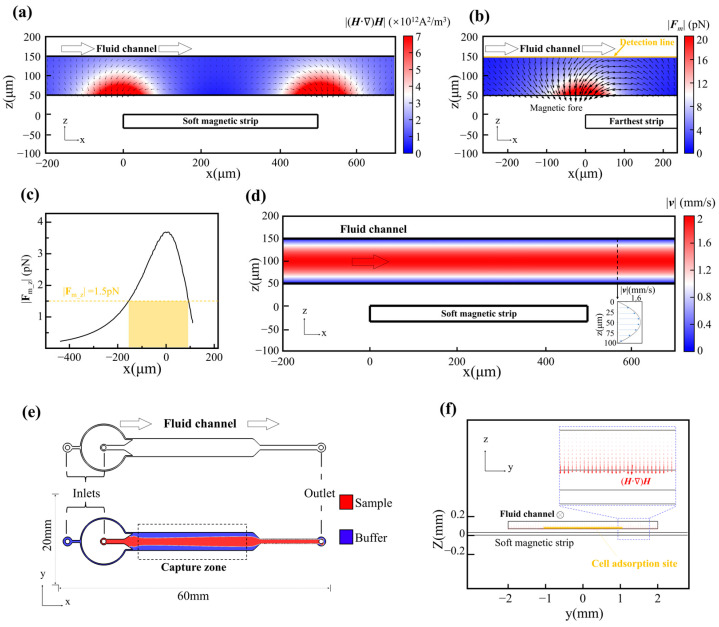
Coupled magnetic and hydrodynamic simulations of the MagSculptor system. (**a**) Spatial distribution of the magnetic force factor (***H***·∇)***H*** across the microchannel. (**b**) Simulated magnetic force profile for a 2.8 µm magnetic bead near the distal strip. (**c**) The vertical force component (z-direction) acting on a 2.8 μm magnetic bead along the yellow detection line in (**b**). (**d**) Flow velocity profile in the channel. (**e**) Phase-field simulation of sample and buffer interface distribution. (**f**) Axial view of combined flow and magnetic field simulation. Red arrows are scaled proportional to (***H***·∇)***H*** and reach their maximum in the capture regions.

To accurately characterize the forces acting on bead-labelled cells within the MagSculptor system, we performed FEM simulations coupling the magnetic and hydrodynamic fields (Section 2.5). In a gradient magnetic field, superparamagnetic beads are first magnetized and then subjected to a net translational force that is proportional to the field-dependent factor (***H***·∇)***H***, as described by Equation (1). The scalar–vector product plotted in Figure 2a therefore serves as a convenient magnetic force factor that summarizes the spatial variation in the force landscape experienced by a particle of fixed magnetic susceptibility.

### 3.3. Simulation of Coupled Magnetic Fields, Flow, and Cell Capture Dynamics

Heatmap visualizations of the magnetic force factor (***H***·∇)***H*** show that each soft-magnetic strip generates a pair of localized force maxima near its lateral edges, consistent with the high-gradient zones predicted by the field-shaping analysis. In this landscape, the vertical component of the magnetic force is particularly critical because it pulls bead-labelled cells downward toward the strip surface and governs the onset of physical contact between cells and the substrate.

To assess capture reliability under worst-case conditions, we modelled the trajectory of a 20 µm-diameter cell conjugated with a single 2.8 µm magnetic bead (Figure 2b). Even along a minimal-force streamline, identified as the least favorable path in the magnetic-force field, simulations show that the cell is still deflected toward the bottom of the channel before exiting the capture region. The profile in Figure 2c confirms that even weakly labelled cells are drawn toward capture sites under realistic combinations of flow rate and field strength.

We next examined the hydrodynamic behavior of the flow field within the microchannel. Simulations confirmed a laminar Poiseuille-type velocity profile along the channel height and a uniform axial flow along its length (Figure 2d). To confine cells within the optimal capture zone above the strip array, a sheath flow was introduced to hydrodynamically focus the sample into the central ~50 µm of the 100 µm-tall channel (Figure 2e,f).

Finally, we incorporated dynamic mechanical interactions into the model to evaluate the interplay between magnetic forces, hydrodynamic drag, and substrate friction. Time-dependent simulations indicated that while magnetic and frictional forces remain essentially stable at a given position, the drag force varies with both local flow velocity and vertical (*z*-axis) location. This behavior highlights the importance of controlling vertical flow confinement to minimize variability in capture efficacy across the strip array.

**Figure 3:**
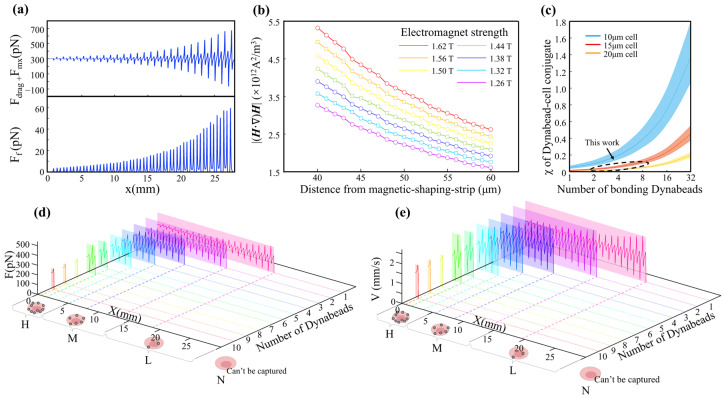
Parametric analysis and dynamic simulation of cell subtype sorting in the MagSculptor system. (**a**) The distribution of the horizontal magnetic force and frictional force brought by a single magnetic bead (2.8 μm) to the cell. (**b**) Magnetic field profiles above the soft magnetic strip as a function of varying electromagnet current and strip-to-channel distance. (**c**) Parametric analysis of effective magnetic susceptibility of bead–cell conjugates. The dashed circular regions delineate the modeling range encompassing the cellular dimensions investigated in this study. (**d**) Force distribution profiles for different cell types within the microchannel. Modeling parameters: cell diameter = 15 μm, magnetic bead diameter = 2.8 μm. (**e**) Simulated velocity and position trajectories for cells with different magnetic labelling densities. Modeling parameters: cell diameter = 15 μm, magnetic bead diameter = 2.8 μm.

To deepen our understanding of cell-sorting dynamics in MagSculptor, we further performed parametric analyses of both the magnetic-field conditions and cell–bead interactions (Figure 3). The representative microchannel used for modelling had a width of 4 mm, a height of 100 µm, and a total sorting-zone length of 27 mm. The horizontal and vertical components of the magnetic force, derived from the spatial gradient of the field, were analyzed separately to clarify their respective roles in capture dynamics (Figure 3a).

The vertical force component determines the contact stability between a bead-labelled cell and the substrate. Once a sufficient downward magnetic force is exerted, frictional interaction dominates and regulates axial migration along the strip. The coefficient of dynamic friction in aqueous environments, assumed to be between 0.05–0.15, is sufficient to ensure that captured cells are gradually slowed and immobilized near the strip surface. In contrast, the horizontal force component exhibits oscillatory behavior across the strip boundary, which can induce micro-accelerations or decelerations of partially captured cells.

To select magnetic-field parameters that are resilient to process-induced variations, we performed FEM simulations in which the electromagnet current and the vertical distance between the microchannel and the soft-magnetic strips were varied systematically. The results show that increasing the magnetic field can compensate for larger strip-to-channel distances (Figure 3b). This tunability allows precise alignment between magnetic-field peaks and the capture zone, even in the presence of fabrication-induced deviations in channel height. Treating cells and their bonded magnetic beads as integrated conjugates, the effective magnetic properties of these entities vary with bead loading. Larger cell volumes attenuate the relative contribution of a fixed number of beads to the overall magnetic response, thereby reducing errors arising from cellular-volume variations. From a statistical perspective, cancer cells tend to have larger average diameters than normal cells, which can be advantageous for gradient-capture architectures.

Previous reports document substantial variability in size measurements even for nominally identical cell types; however, MDA-MB-231, MCF-7, and A549 cells consistently fall within the 15 ± 3 µm range [30,31,32], whereas Caco-2 cells are larger and exhibit broader size distributions (around 20 µm) [33,34,35]. Although Caco-2 cells show considerable size dispersion, for these larger cells, the influence of magnetic bead content on the magnetic properties of the cell–bead conjugate becomes relatively attenuated (Figure 3c). This effect serendipitously enables predictable trajectory modelling for Caco-2 cells despite their heterogeneous size distribution. To ensure comprehensive predictive accuracy, kinematic simulations therefore covered the full cell-size spectrum from 15 µm to 20 µm.

The terminal state of cells within the microchannel corresponds to stationary positions at specific locations where the balance among fluid drags, magnetic forces, and friction yields zero net force along the direction of motion. Resultant-force components along the flow direction can be plotted as line profiles (Figure 3d) in which positions with zero horizontal net force denote stable stationary states. Capture trajectories for cells with nominal 20 µm diameters were also modelled, explicitly accounting for larger cells such as Caco-2 (Figure S3a). To dynamically describe the motion of different cell subtypes, we integrated spatially resolved flow and magnetic fields with inlet and outlet boundary conditions and used a forward-Euler scheme (Δt = 1 × 10^−5^ s) to update state variables over time. Velocity–displacement traces for different cell types were then extracted to generate the plots in Figure 3e. As cells encounter magnetic capture barriers, their velocities change under the combined influence of magnetic and frictional forces, increasing the velocity differential between the cells and the surrounding fluid. Alternating acceleration and deceleration induce oscillatory velocity behavior whose amplitude grows with barrier strength, eventually decaying to zero at the capture position.

For Caco-2 cells, which possess characteristically larger volumes, we used a 20 µm diameter in simulations to represent their sorting dynamics. Because the increased cellular volume effectively dilutes the magnetic influence of individual beads, these larger cells consistently exhibit capture sites that lag by approximately 2–3 strip positions relative to smaller counterparts (Figure S3b). This size-dependent capture behavior reflects the fundamental relationship between cellular volume and effective magnetic susceptibility: larger cells require proportionally higher bead densities to achieve capture efficiencies comparable to smaller cells within the engineered magnetic-gradient landscape.

By correlating magnetic bead load with capture position, cell subtypes can thus be grouped into four ordered phenotypes—H (High), M (Medium), L (Low), and N (Negative, uncaptured). Dynamic capture positions closely track the static equilibrium positions but exhibit slight, predictable downstream shifts due to cellular inertia. Importantly, this inertial effect does not propagate across an entire strip, thereby preserving the positional resolution necessary for reliable subtype assignment. Overall, the combined magnetic–hydrodynamic field architecture in MagSculptor provides reproducible separation performance with subpopulation-level resolution across the four EpCAM-defined subgroups and offers practical guidance for choosing operating parameters in experiments.

### 3.4. Subtype Isolation of Cells

**Figure 4:**
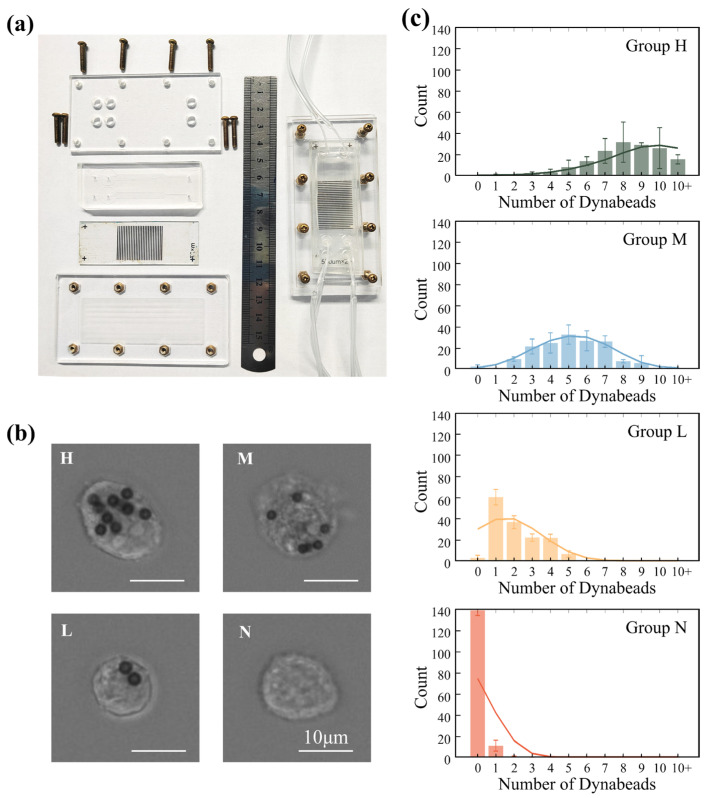
Experimental implementation and validation of MagSculptor in cell subtype sorting. (**a**) Photograph of the MagSculptor assembly. A dual-channel configuration is shown as an example, demonstrating that the platform can be extended to parallel channels for increased overall throughput. (**b**) Representative bright-field images of MDA-MB-231 cells in the four MagSculptor-defined subtypes (H, M, L, and N). Cells were imaged after sorting to illustrate typical bead-labelling patterns in each subgroup (scale bars, 10 µm). (**c**) Bar graph depicting the average magnetic bead occupancy per cell across the four sorted groups (H, M, L, and N) for MDA-MB-231 cells. For each subtype and experiment, 150 cells were randomly selected from three non-overlapping fields of view (450 cells per subtype across three independent sorting runs) and manually scored for bead number per cell. Bars indicate mean values and error bars represent standard deviation (mean ± s.d.).

Using the assembled MagSculptor chip described in Section 2.4 (Figure 4a), we next evaluated its ability to resolve EpCAM-defined subtypes within four epithelial cancer cell lines (Caco-2, MCF-7, MDA-MB-231, and A549). For each experiment, a single cell line was incubated with anti-EpCAM magnetic microbeads and then introduced into the device under identical magnetic and flow conditions; the four cell lines were processed sequentially on the same chip to ensure direct comparability while avoiding cross-sample interference. Magnetically labelled cells were retained on the strip array according to bead load, whereas unlabeled cells were washed to the outlet. Captured cells were therefore partitioned into high-, medium-, and low-bead subtypes (H, M, and L), while the downstream effluent comprised the negative (N) fraction. The surface-bound bead numbers ranged from several to ~10 beads per cell in the H group and approached zero in the N group (Figure 4b).

To visualize how each cell line distributes across the graded magnetic landscape, we quantified, for every stripe (27 stripes in total), the number of captured cells in the stripe-array images shown in Figure S4a and plotted stripe-wise histograms in Figure S4b and Table S1. This analysis converts the static images into spatial subtype profiles along the *x*-axis of the device. Consistent with established EpCAM phenotypes, Caco-2 displays the most upstream (high-bead) capture pattern, followed by MCF-7 and MDA-MB-231, with A549 showing the most downstream (low-bead) distribution, in agreement with previous reports [36,37,38]. We also note that, due to intrinsic heterogeneity, a small fraction of MDA-MB-231 cells occupy upstream stripes with higher apparent EpCAM level—an inter-individual variation that MagSculptor can make accessible for downstream mechanistic studies. To substantiate the semi-quantitative link between bead-defined subtypes and EpCAM abundance, we performed Western blotting on MCF-7 subgroups (H/M/L/N); β-actin-normalized EpCAM levels decreased monotonically from H → M → L → N, in line with the device-based subtype ranking (Figure S5).

Following separation, cell positions within the microchannel were imaged by fluorescence microscopy. Representative bright-field images of the four MagSculptor-defined subtypes for MDA-MB-231 cells are shown in Figure 4b. Pseudo-color mapping was applied only in post-processing to distinguish the four cell lines in the stripe-array images of Supplementary Figure S4a, enabling precise positional analysis relative to the soft-magnetic strip array while preserving the fact that each line was run in an independent experiment. Along the progressive magnetic gradient from strip 1 to strip 27, strongly labelled cells are retained on earlier stripes, whereas weakly labelled cells migrate further downstream and accumulate near the outlet. By grouping stripes 1–9, 10–18, and 19–27 as H, M, and L regions, respectively, we obtained distribution patterns consistent with the simulated bead-load ranges of approximately 6–10, 3–5, and 1–2 beads per cell (Figure 3e). To quantify separation performance, we report two metrics. Overall collection (overall recovery)—the ratio of H+M+L+N cells to the input—assesses chip-induced loss and is phenotype-independent; for MDA-MB-231, this value reached 96.4 ± 1.1% (*n* = 3). Positive capture rate, defined over H/M/L only, reflects how efficiently EpCAM-positive cells are partitioned into resolved subtypes and is analyzed in subsequent sections; in whole-blood spike-in experiments, we apply the same concept but normalize to the known number of spiked tumor cells (Section 3.4).

We randomly selected 150 cells from each MagSculptor-defined subgroup and quantified the number of surface-bound beads per cell. Statistical analysis confirmed significant differences in bead load between adjacent subtypes (Figure 4c), and the overall distributions exhibited a monotonic decrease in bead number from H to N. Because intrinsic cell-to-cell size variability modulates hydrodynamic drag, the experimental bead-count histograms are broader than the idealized trajectories predicted by simulations. In practice, separation performance can be tuned to the size profile of a given sample by modestly increasing the magnetic field and/or lowering the flow rate for larger cells, and doing the converse for smaller cells. For samples with very broad size distributions, an upstream size-harmonization step (e.g., micro-sieve or size-based microfluidic selection) can further stabilize subtype boundaries without requiring any modification to the core MagSculptor architecture.

To benchmark MagSculptor against a widely used standard, we performed parallel experiments focusing on low-phenotype subtype discrimination and compared our results with flow cytometry. For high EpCAM-expressing MCF-7 cells, fluorescence-activated cell sorting (FACS) readily resolved positive populations from negative controls. In contrast, for low-EpCAM MDA-MB-231 cells, strong cellular autofluorescence produced fluorescence signals that were nearly indistinguishable from those of antibody-labelled cells, making positive/negative classification unreliable and effective sorting essentially infeasible (Figure S6). Under the same labelling conditions, MagSculptor not only distinguished positive from negative MDA-MB-231 populations but also further partitioned the positive cohort into H/M/L subtypes. Across three independent sorting runs, cell viability remained between 98.5% and 100.0% across all fractions (*n* = 3) (Figure S7), with no statistically significant differences between pre-sorted and post-sorted samples (two-tailed Student’s *t*-test, *p* > 0.05), indicating that microfluidic sorting does not measurably impair viability. Moreover, doxorubicin dose–response assays on sorted MCF-7 subgroups showed progressive right-shifts in the viability curves (L > M > H in IC_50_), demonstrating that, within this MCF-7 model, lower-EpCAM phenotypes exhibit greater drug tolerance (Figure S8) and illustrating how subtype-resolved fractions can support downstream functional readouts in vitro [39,40].

Within the broader landscape of magnetophoretic and immunomagnetic microfluidic platforms, MagSculptor is best viewed as a complementary front-end rather than a replacement for existing technologies. Representative devices summarized in Supplementary Table S2 illustrate several distinct design directions and the associated trade-offs between throughput, architectural complexity, and fractionation dimensionality [26,41,42,43,44,45,46,47,48,49,50]. High-throughput MACS-inspired or ferrohydrodynamic systems typically operate at the mL·h^−1^ scale but collapse all labelled cells into a single positive fraction, providing efficient binary enrichment without resolving intra-line heterogeneity, whereas other magnetophoretic architectures introduce multiple fractionation bins or multi-stage cascades at the cost of more intricate microstructures or lower per-bin throughput. In this context, the present MagSculptor implementation represents one possible compromise, achieving four discrete expression-resolved fractions at ~4 mL·h^−1^ in a single straight channel without internal obstacles. These comparisons are not intended as a ranking of “better” or “worse” platforms, but rather as a way to clarify the niche that MagSculptor occupies within the existing design space.

To probe the performance of MagSculptor under clinically relevant backgrounds, we next examined EpCAM-based subtype capture from spiked whole blood. Four independent 4 mL aliquots of whole blood were each spiked with approximately 100, 1000, 10,000, or 100,000 MDA-MB-231 cells and incubated with anti-EpCAM magnetic beads at 1.2–1.4 × 10^6^ beads/mL for 30 min at room temperature. The Calcein label, present only on the spiked tumor cells and not on endogenous blood cells, was used to identify tumor cells in both the input suspensions and the captured fractions. Considering the higher viscosity of whole blood relative to culture medium and the additional drag from dense erythrocytes, we set the sample flow to 2500 µL/h and the buffer flow to 800 µL/h and achieved stable subtype-gradient capture of cancer cells from whole blood (Figure S9). These conditions correspond to a total flow rate of 3300 µL/h (sample-to-buffer ratio ≈ 3.1:1), an average axial velocity of ≈2.3 mm/s in the 4 mm × 100 µm channel, and an estimated residence time of ≈12 s across the 27 mm-long strip array. Under this flow configuration, processing a full 4 mL aliquot of whole blood requires approximately 1.2 h of run time per sample. Because the spiked MDA-MB-231 population is not uniformly EpCAM-positive, cells that failed to bind beads remained dispersed in the blood matrix. Accordingly, in whole-blood experiments, we defined the positive capture rate as the fraction of spiked tumor cells that were retrieved on the capture strips as EpCAM-positive, i.e., the number of Calcein-positive cells captured in the H, M, and L fractions divided by the known number of Calcein-positive cells spiked into each 4 mL aliquot. Under these conditions, 22.4–35.6% (*n* = 4) of spiked MDA-MB-231 cells were captured as EpCAM-positive. Because endogenous blood cells were not fluorescently labelled, occasional bead binding to host cells, if present, would not contribute to the numerator and can only decrease the measured positive capture rate. In parallel, using the same chip and operating conditions, Caco-2 spike-in experiments yielded markedly higher positive capture rates of 71.3–83.9% (*n* = 4), consistent with their higher EpCAM expression. Even at very low spiking densities (<25 cells/mL), Caco-2 cells were captured as isolated single cells along the strip array, enabling direct visualization of subtype-resolved capture positions. Together, these results demonstrate that MagSculptor can operate reliably in complex whole-blood environments while preserving subtype information across a broad range of marker expression levels and input cell concentrations (Figure S9).

## 4. Conclusions

In this study, we introduced MagSculptor, a microfluidic platform that uses soft-magnetic field shaping and laminar flow control to fractionate low-expression cell subtypes. By integrating arrays of annealed amorphous 1J85 nickel–iron soft-magnetic strips with a side-mounted, tunable electromagnet, MagSculptor sculpts localized high-gradient zones within a straight microchannel and converts modest differences in magnetic bead loading into distinct capture positions. FEM simulations of coupled magnetic and hydrodynamic fields guided the choice of strip–channel spacing, field strength, and flow conditions, and established the operating window within which bead-labelled cells can be reproducibly captured and assigned to High (H), Medium (M), Low (L), and Negative (N) subgroups.

Experimentally, we validated MagSculptor using four epithelial cancer cell lines with different EpCAM expression levels. Under identical labelling, magnetic, and flow conditions, each cell line was processed in separate runs on the same device, yielding reproducible subtype distributions that reflect their known EpCAM phenotypes and reveal graded heterogeneity even in low-EpCAM lines where conventional flow cytometry becomes unreliable. Bead-count statistics confirmed monotonic decreases in magnetic labelling from H to N, Western blotting showed a corresponding decline in EpCAM abundance, and doxorubicin dose–response assays on sorted MCF-7 subgroups demonstrated expression-dependent differences in drug sensitivity. Cell viability remained above 95% across all fractions, and spike-in experiments with whole blood showed that MagSculptor can operate reliably in complex blood matrices while preserving subtype information, providing an initial indication of compatibility with liquid-biopsy-like samples.

Taken together, these results indicate that MagSculptor provides a practical extension of traditional immunomagnetic separation from binary positive/negative enrichment toward quantitative, multi-level isolation of low-expression subtypes within a single immunomagnetically labelled population. The device is compatible with standard bead-labelling protocols, relies on a simple and modular chip architecture, and requires only adjustment of magnetic field strength and flow rate to accommodate different cell sizes and sample viscosities. In this regard, MagSculptor is intended to complement rather than replace existing MACS and flow-cytometric platforms. The survey of representative systems, summarized in Supplementary Table S2, highlights how our implementation occupies a region of design space that trades extreme throughput and complex internal microstructures for intermediate-throughput, four-level fractionation with straightforward fraction collection. In a front-end role, MagSculptor can therefore serve as one possible module for downstream single-cell omics, functional assays, and drug-response profiling, particularly in settings where subtle shifts in surface-marker expression carry biological or prospective clinical significance.

Several limitations of the present study should be noted. These also delineate natural directions for future development. First, all subtype-fractionation experiments were performed in a sequential manner, processing one immunomagnetically labeled population per run rather than performing simultaneous multi-line or multi-marker sorting. Second, MagSculptor fundamentally relies on antibody-mediated bead labeling and the availability of suitable surface markers; subtypes defined by markers that are absent, inaccessible, or poorly labeled cannot be resolved, and the effective sensitivity of the system is ultimately constrained by the immunomagnetic chemistry. Third, in this work, we defined an operational detection limit in terms of the minimum bead load per cell that still yields capture under given flow and field conditions rather than a fully calibrated antigen copy-number threshold. Finally, the biological validation in this work was restricted to cultured epithelial cancer cell lines and controlled spike-in experiments in whole blood from healthy donors and thus represents a preclinical proof-of-concept rather than a validated clinical assay. In particular, our current data do not yet address analytical performance in true liquid-biopsy settings, where circulating tumor cells are extremely rare and blood composition varies across patients and treatment regimens. Additional studies with rare-cell spike-ins at clinically realistic abundances, patient-derived circulating tumor cells, and longitudinal clinical samples will be required to assess robustness to blood components and to define how MagSculptor could be integrated into future clinical decision-making workflows.

Future developments may therefore focus on extending the MagSculptor concept along these axes: incorporating additional biomarkers and bead chemistries, integrating multiplexed channels and automated fraction collection to enable parallel processing, and coupling the platform with quantitative calibration workflows (e.g., flow cytometry or imaging-based standards) to establish absolute expression limits of detection in copy-number units. In parallel, applying MagSculptor to primary tumor samples, circulating tumor cells, or immune-cell subsets will be important to test its robustness in clinically relevant settings and to explore how expression-resolved subpopulations relate to functional heterogeneity in drug response or immune interactions.

## Data Availability

The original contributions presented in this study are included in the article. Further inquiries can be directed to the corresponding authors.

## Supplementary Materials

The following supporting information can be downloaded at: https://www.mdpi.com/article/10.3390/bios16010041/s1. Method S-M1: FEM model and particle-trajectory simulations; Method S-M2: Additional device tolerances, assembly, and surface conditioning; Method S-M3: Quantification of bead-load distributions on labelled cells; Figure S1: Microfabricated soft-magnetic strips on substrate and local magnifications; Figure S2: Operational workflow and experimental setup; Figure S3: Parametric analysis and dynamic simulation of cell subtype sorting in the MagSculptor system; Figure S4: Stripe-resolved capture patterns and statistical distributions of EpCAM-defined subtypes for four epithelial cancer cell lines; Figure S5: Western blot validation of EpCAM across MagSculptor-sorted MCF-7 subgroups; Figure S6: Flow cytometric assessment of EpCAM expression in MCF-7, MDA-MB-231, Caco-2 and A549 cell lines; Figure S7: Assessment of cell viability following chip-based sorting operations (*n* = 3); Figure S8: Doxorubicin response of MagSculptor-sorted MCF-7 subtypes; Figure S9: Capture of MDA-MB-231 cells from whole blood samples; Table S1: Stripe-wise capture statistics for four epithelial cancer cell lines, each loaded at approximately 1000 cells per run in a single representative experiment (*n* = 1 independent capture run per line; the number of analyzed cells is given in the “Captured cells (number)” row); Table S2: Representative magnetophoretic, ferrohydrodynamic and immunomagnetic microfluidic platforms for rare cell separation and phenotypic/antigen-level profiling.

## Abbreviations

The following abbreviations are used in this manuscript:

EpCAM	Epithelial cell adhesion molecule
PBS	Phosphate-buffered saline
MACS	Magnetic-activated cell sorting
FACS	Fluorescence-activated cell sorting
CTC	Circulating tumor cell
FEM	Finite element method
PMMA	Polymethyl methacrylate
PC	Polycarbonate
CAR-T	Chimeric Antigen Receptor T-Cell Immunotherapy
