# Supplementary material for: MagSculptor: A Microfluidic Platform for High-Resolution Magnetic Fractionation of Low-Expression Cell Subtypes
Source: Biosensors (Basel). 2026 Jan 4;16(1):41. doi: 10.3390/bios16010041 (PMC12838995; doi:10.3390/bios16010041)
Supplement: Supplementary file 1 [file biosensors-16-00041-s001.zip › biosensors-4035853-supplementary.pdf]

biosensors

MDPI

Supplementary Materials

MagSculptor: A Microfluidic Platform for High-Resolution Magnetic Fractionation of Low-Expression Cell Subtypes

Zhenwei Liang [1,2,†], Yujiao Wang [1,2,†], Xuanhe Zhang [1,2], Yiqing Chen [1,2], Guoxu Yu [1,2], Xiaolei Guo [1,2,3], Yuan Ma [1,2,*] and Jiadao Wang [1,2,*]

[1] Department of Mechanical Engineering, Tsinghua University, Beijing 100084, PR China
[2] State Key Laboratory of Tribology, Tsinghua University, Beijing 100084, China
[3] Center for Medical Device Evaluation, National Medical Products Administration, Beijing 100081, PR China
[*] Correspondence: yuanma@tsinghua.edu.cn (Y. M.); jdwang@mail.tsinghua.edu.cn (J. W.)
[†] These authors contributed equally to this work.

Supplementary Method S-M1. FEM model and particle-trajectory simulations

S-M1.1 Geometry and physics interfaces

The finite element method (FEM) simulations were implemented in COMSOL Multiphysics® (AC/DC and CFD modules) to resolve the coupled magnetic and hydrodynamic fields in the MagSculptor chip. The model domain included the soft-magnetic strip array (1J85 alloy), the glass substrate, the microchannel (4 mm wide, 100 μm tall), and the surrounding air region, together with the electromagnet core and coil.

Magnetic fields were solved using the Magnetic Fields (mf) interface, while incompressible flow in the microchannel was solved using the Laminar Flow (spf) interface. Where the sample/buffer interface was explicitly illustrated, we additionally used the Two-Phase Flow, Phase Field (tpf) interface to track the interface between the two co-flowing streams.

S-M1.2 Boundary conditions

Magnetic field: At interfaces between the soft-magnetic strips and surrounding air or glass, standard continuity conditions for the magnetic flux density ***B*** and magnetic field ***H*** were applied. The outer boundaries of the simulation domain were set to a magnetically insulating condition, which approximates a closed magnetic circuit far from the microchannel. The electromagnet coil was represented by a homogenized current density corresponding to the experimentally applied excitation; the resulting background field magnitude along the strip array was matched to the measured values reported in the main text.

Fluid flow: For PBS-based subtype-fractionation experiments, the sample and buffer inlets were modelled as laminar inflow boundaries with prescribed volumetric flow rates corresponding to the experimental conditions. In standard subtype-sorting experiments, the sample flow rate was set to 3000 μL/h and the buffer flow rate to 1000 μL/h, giving a total flow rate of 4000 μL/h. For whole-blood spike-in simulations, we used flow rates of 2500 μL/h for the blood sample and 800 μL/h for the buffer stream, consistent with the conditions used in the experiments described in Section 3.4. The outlet was set to a zero-pressure boundary ($p = 0$ Pa), and all channel walls were treated as no-slip boundaries.

S-M1.3 Material properties

 https://doi.org/10.3390/xxxxx

The soft-magnetic strips were fabricated from 1J85 alloy and modelled as a nonlinear soft ferromagnetic material. The B–H curve was approximated from supplier data and characterized by the following key parameters: a differential permeability near the origin corresponding to μ ≈ 0.08, inductances of approximately 112.5 mH/m and 377.0 mH/m in different regions of the magnetization curve, coercive field H_c ≈ 0.42 A/m, saturation flux density B_s ≈ 0.75 T, and remanent flux density Br ≈ 0.26 T. These values were used to define the nonlinear relative permeability as a function of $|\boldsymbol{H}|$.

The microchannel fluid was treated as an incompressible Newtonian fluid. For PBS-based runs, both the sample and buffer phases were assigned water-like properties with a density of ϱ = 1000 kg·m^{-3} and a dynamic viscosity of η = 1.0 × 10^{-3} Pa·s at room temperature. For whole-blood simulations, the sample phase was assigned typical whole-blood properties with a density of ϱ = 1050 kg·m^{-3} and dynamic viscosity of η = 3.5 × 10^{-3} Pa·s, whereas the buffer stream retained the water-like values given above.

Magnetic beads were modelled as superparamagnetic particles with a magnetization behavior similar to magnetite (Fe_3O_4), and their effective magnetization was calculated from the local magnetic field assuming a linear response below saturation. In the particle-level calculations described below, the effective magnetic moment m of a bead-labelled cell was updated based on the local field strength, consistent with this magnetization model.

S-M1.4 Meshing and convergence criteria

To resolve the strong field gradients near the strip edges and at the channel–strip interface, we used a mesh with progressive refinement near all material interfaces and geometric corners. In particular, we applied boundary-layer and corner refinement along the strip edges and at the channel floor and ceiling, with a minimum element size on the order of 0.05 μm in the regions of highest curvature. The mesh size was gradually increased in the bulk of the channel and in the surrounding air domain to reduce computational cost.

Mesh-independence was verified by performing simulations with successively refined meshes and monitoring key output quantities, such as the peak magnitude of the field-gradient term $|\nabla|\boldsymbol{B}|^2|$ at a height of 50 μm above the strip array and the integrated magnetic force acting on a model bead-labelled cell. Further mesh refinement changed these quantities by less than 2%, indicating that the chosen mesh was sufficient for accurate evaluation of the forces relevant to cell capture.

For the steady-state magnetic and flow fields, the stationary solver in COMSOL was used with absolute and relative residual tolerances tightened to <10^{-5}. Convergence was confirmed by ensuring that the residuals fell below these thresholds and that further tightening of tolerances did not alter the fields within numerical precision.

S-M1.5 Particle-trajectory simulations (MATLAB implementation)

To translate the FEM results into bead-load-dependent cell trajectories along the strip array, we coupled the COMSOL-derived field gradients to a custom MATLAB script implementing forward-Euler integration of the equations of motion for a bead-labelled cell. The numerical implementation is summarized here in sufficient detail to allow for independent reproduction.

We first exported one-dimensional profiles of the magnetic field-gradient term $(\boldsymbol{H}\cdot\nabla)\boldsymbol{H}$ along the strip direction at a fixed vertical height (corresponding to the mid-plane of the microchannel) from COMSOL into text files. These files contain the spatial coordinate x and the corresponding horizontal and vertical components of $(\boldsymbol{H}\cdot\nabla)\boldsymbol{H}$. In MATLAB, we read these profiles and constructed interpolation functions f_Hx(x) and f_Hy(x) using linear interpolation with extrapolation at the boundaries.

Each bead-labelled cell was modelled as a rigid sphere of effective diameter D_{pc} corresponding to the combined volume of the cell (diameter D_c = 20 μm) and n_p attached beads (bead diameter D_p = 2.8 μm). The total mass m of the composite particle was computed from the densities of the cell (ρ_c = 1000 kg/m^3) and beads (ρ_p = 5,180 kg/m^3). The effective magnetic parameter $\tilde{\mu}$ was calculated as:

$$\tilde{\mu} = n_p \mu_0 (\frac{4}{3}) \pi (\frac{D_p}{2})^3 (\frac{3\chi}{\chi + 3}) \tag{S1}$$

where μ_0 is the vacuum permeability and the factor $(3\chi/(\chi+3))$ reflects the estimated susceptibility of the bead material under the applied fields ($\chi \approx 10$).

The horizontal component of the magnetic force acting on the bead-labelled cell at position x was then computed as:

$$F_{mx}(x) = \tilde{\mu} \cdot ((\boldsymbol{H} \cdot \nabla)\boldsymbol{H}(x) \cdot \boldsymbol{i}_x) \tag{S2}$$

where $(\boldsymbol{H}\cdot\nabla)\boldsymbol{H}(x)\cdot\boldsymbol{i}_x$ is obtained from f_Hx(x). A friction-like term $F_{tri} = \tilde{\mu}\,(\boldsymbol{H}\cdot\nabla)\boldsymbol{H}(x)\cdot\boldsymbol{i}_y$, μ_f was included to represent additional resistance associated with particle–surface interactions, where μ_f is an empirical friction coefficient. If F_{tri} became negative, it was set to zero.

The hydrodynamic drag force was modelled by Stokes' law:

$$F_{drag} = 3\pi D_{pc} \eta (v_0 - v) \tag{S3}$$

where η is the dynamic viscosity of the fluid, v_0 is the local fluid velocity (taken as the steady-state flow velocity at the particle position obtained from the COMSOL laminar-flow solution), and v is the instantaneous particle velocity. The net horizontal force was then:

$$F_x = F_{mx} - F_{tri} + F_{drag} \tag{S4}$$

The equations of motion were integrated in time using an explicit forward Euler scheme with a time step $\Delta t = 10^{-5}$ s. At each time step, position and velocity were updated according to:

$$x_{k+1} = x_k + v_k \Delta t \tag{S5}$$

$$v_{k+1} = v_k + (F_x(x_k, v_k))\Delta t \tag{S6}$$

starting from an initial position x_0 = –27.49 mm and initial velocity $v_0 = 1.2 \times 10^{-3}$ m/s. The integration proceeded until one of the following stopping criteria was met: (i) $|v| < 10^{-6}$ m/s (particle effectively stationary), (ii) $x > 0.5$ m (particle traversed the entire strip array), or (iii) $x < -27.5$ mm (particle moved out of the domain). For each chosen bead number n_p, we simulated the corresponding trajectory and recorded the final position and velocity, as well as the full x–$v(x)$ curve (Figure 3f).

For consistency with the coordinate convention used in the main text, all simulated x positions were shifted by +27 mm when reporting capture locations, so that the origin aligns with the entrance of the strip array as shown in the main figures.

Supplementary Method S-M2. Additional device tolerances, assembly, and surface conditioning

S-M2.1 Geometric tolerances and strip–channel gap

The nominal device geometry (soft-magnetic strip thickness, width and pitch, strip length, and channel width/height) is described in Sections 2.3 and 2.4 of the main text. Here, we summarize the additional tolerances and assembly-related details that are most relevant for reproducing the magnetic field distribution and cell–surface interactions.

The strip array was patterned from 35 μm-thick 1J85 on glass as described in Section 2.3. Within a given batch, the fabricated strip width remained within ±10% of the 500 μm design, and profilometry confirmed that the strip thickness was close to the nominal 35 μm value along the channel length. These tolerances were used directly in the FEM model in Supplementary Method S-M1.

The microchannel was implemented as a three-layer polycarbonate (PC) stack (top port plate, 100 μm spacer, and bottom film) as outlined in Section 2.4. The separation between the soft-magnetic strips and the flow, denoted D, is defined by the thickness of the bottom PC film. The nominal thickness of this film was 50 μm, and profilometry measurements at multiple locations yielded a value of 50 ± 3 μm. This ±3 μm range therefore represents the uncertainty in the strip–channel gap **D** in the physical device.

To evaluate the impact of this tolerance on magnetic capture, we performed a FEM-based sensitivity analysis by varying D within the measured range and recomputing the magnetic forces acting on a bead-labelled cell at the capture height (see S-M1). Across this range, the magnitude of the computed magnetic force changed by less than 5%, indicating that the tolerance of the 50 μm PC film has only a minor effect on the field gradients relevant for cell capture under the operating conditions used here.

S-M2.2 Bonding, clamping, and surface conditioning

The three PC layers were bonded by thermal pressing to form a sealed microchannel and to prevent leakage. The PMMA plates were then used to clamp the PC microchannel tightly onto the soft-magnetic strip array, ensuring a well-defined and stable distance between the flowing cells and the underlying strips.

Before each experiment, the assembled microchannel was conditioned by flushing with 1% (w/v) Pluronic F-127 in PBS for 30 min, followed by a PBS rinse, to reduce non-specific adhesion of cells and magnetic beads to the PC and glass surfaces. Together, the defined strip–channel gap **D**, the mechanically stable, thermally bonded PC channel clamped against the strip array, and the Pluronic-based surface treatment define the cell–surface environment experienced by cells in MagSculptor provide the relevant context for interpreting the FEM and experimental results.

https://doi.org/10.3390/xxxxx

Supplementary Method S-M3. Quantification of bead-load distributions on labelled cells

To quantify the distribution of magnetic bead load per cell within each MagSculptor-defined subtype, we performed image-based manual counting on labelled cell suspensions. After immunomagnetic labelling (Section 2.2), an aliquot of each sorted fraction (H, M, L, and N) was adjusted to approximately 5×10^5 cells/mL in PBS. A 10 μL drop of the suspension was deposited onto a clean glass slide and covered with a coverslip to form a monolayer suitable for microscopy.

For each subtype and experiment, three non-overlapping fields of view were identified under a 4× objective. Within these fields, we randomly defined rectangular ROIs that collectively contained 150 cells in total. The corresponding ROIs were then imaged at 10× magnification for bead-load quantification.

All cells within each ROI were manually inspected on the recorded 10× images, and the number of discrete surface-bound beads per cell was recorded. This procedure yielded 150 cells per FOV and 450 cells per subtype per independent sorting run; across three independent runs for MDA-MB-231, this corresponded to a total of 1,350 cells per subtype. Cells carrying more than 10 beads were grouped into a single "≥10 beads" category. The resulting histograms of bead occupancy per cell were used to compute the mean bead load reported in Figure 4c and to construct the subtype-resolved distributions shown in Supplementary Figure S4.

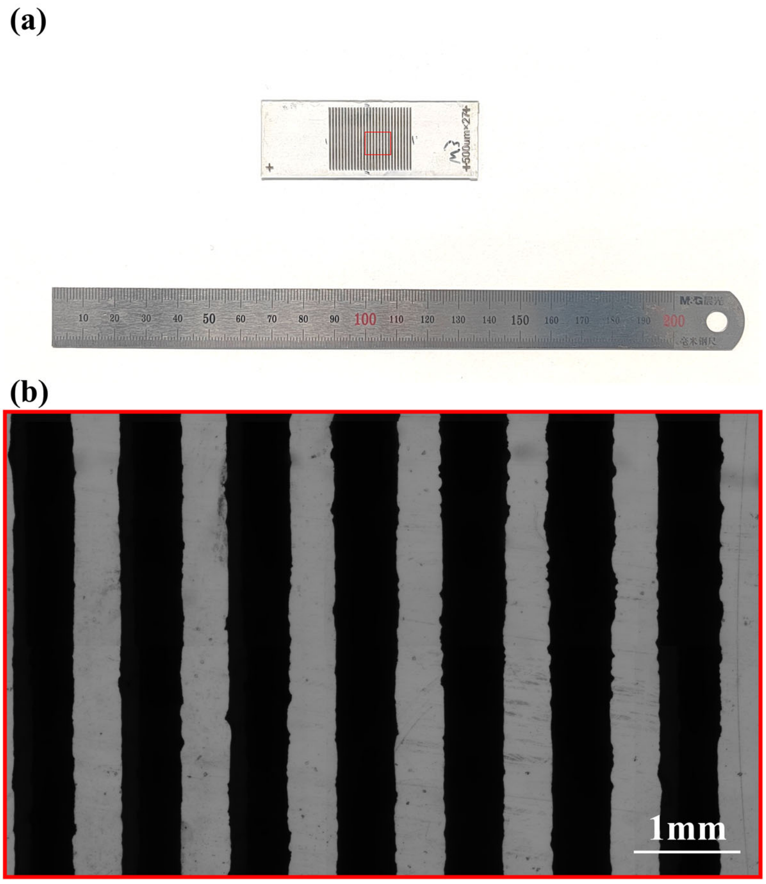

Figure S1. Microfabricated soft-magnetic strips on substrate and local magnifications. (**a**) Overview photograph of the etched 1J85 strip array on a glass substrate (with scale bar). (**b**) Representative magnified microscope images of local regions.

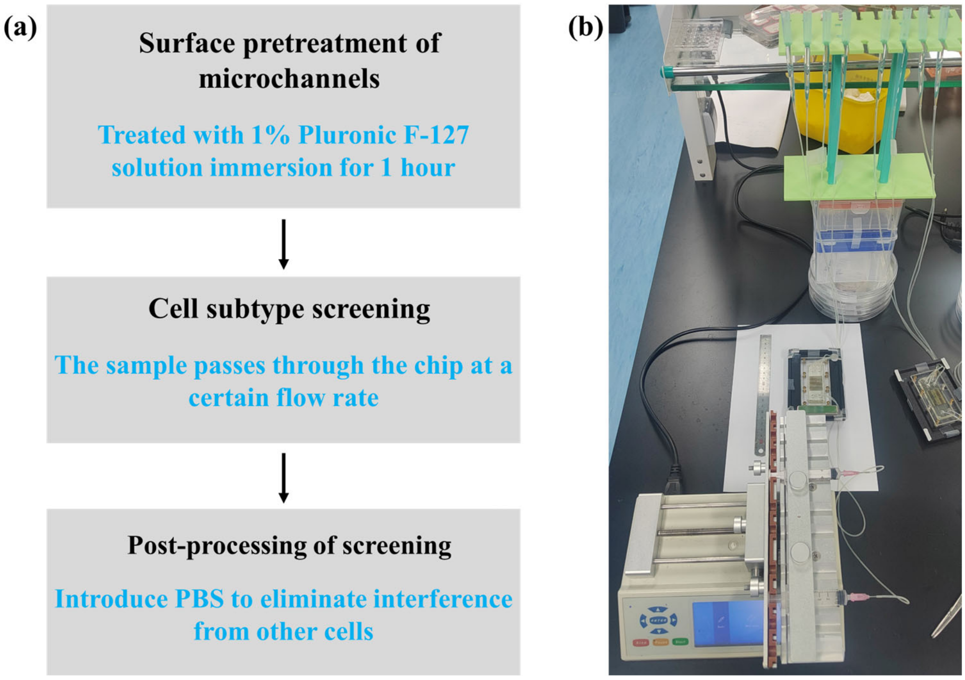

Figure S2. Operational workflow and experimental setup. (**a**) Annotated workflow for MagSculptor subtype sorting: surface conditioning → sample/bead incubation → co-flow focusing (sample 3000 μL/h, 1× PBS 1000 μL/h) → controlled ramp for collection → outlet harvesting of H/M/L/N fractions. An additional ~200 μL of PBS is flushed at the original flow rates after sample loading to eliminate non-magnetic residuals. (**b**) Photograph of the macroscopic setup (with scale bar) showing the suspended pipette-tip reservoir for bubble-free loading and hydrostatic-head tuning, the inlet/outlet connections, and the device under the side magnet/electromagnet.

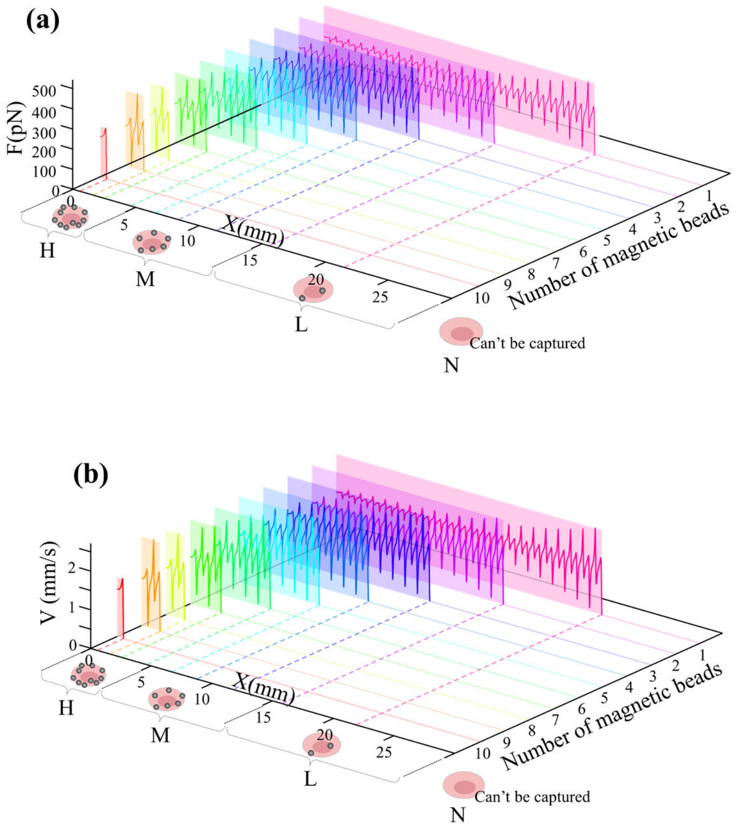

Figure S3. Parametric analysis and dynamic simulation of cell subtype sorting in the MagSculptor system. (**a**) Streamwise magnetic-force profiles for bead-labelled cells at different positions within the microchannel. Each colored curve corresponds to a cell population with a distinct bead load (i.e., a different magnetophoretic mobility). For each bead load, the curve shows the horizontal magnetic force experienced by a cell as a function of its streamwise position along the strip array, indicating whether the net force at a given location tends to pull the cell further downstream or oppose its motion. Modelling parameters: cell diameter = 20 μm; magnetic bead diameter = 2.8 μm. (**b**) Simulated trajectories in the position–velocity (x–v) phase plane for cells with different magnetic labelling densities. As in (a), each colored curve represents a cell population with a specific bead load. The curves illustrate how the streamwise velocity of a cell evolves as it migrates along the channel under the combined action of hydrodynamic drag and magnetic force. Capture occurs when the axial velocity approaches zero, at which point, the cell comes to rest at the corresponding position. Modelling parameters: cell diameter = 20 μm; magnetic bead diameter = 2.8 μm.

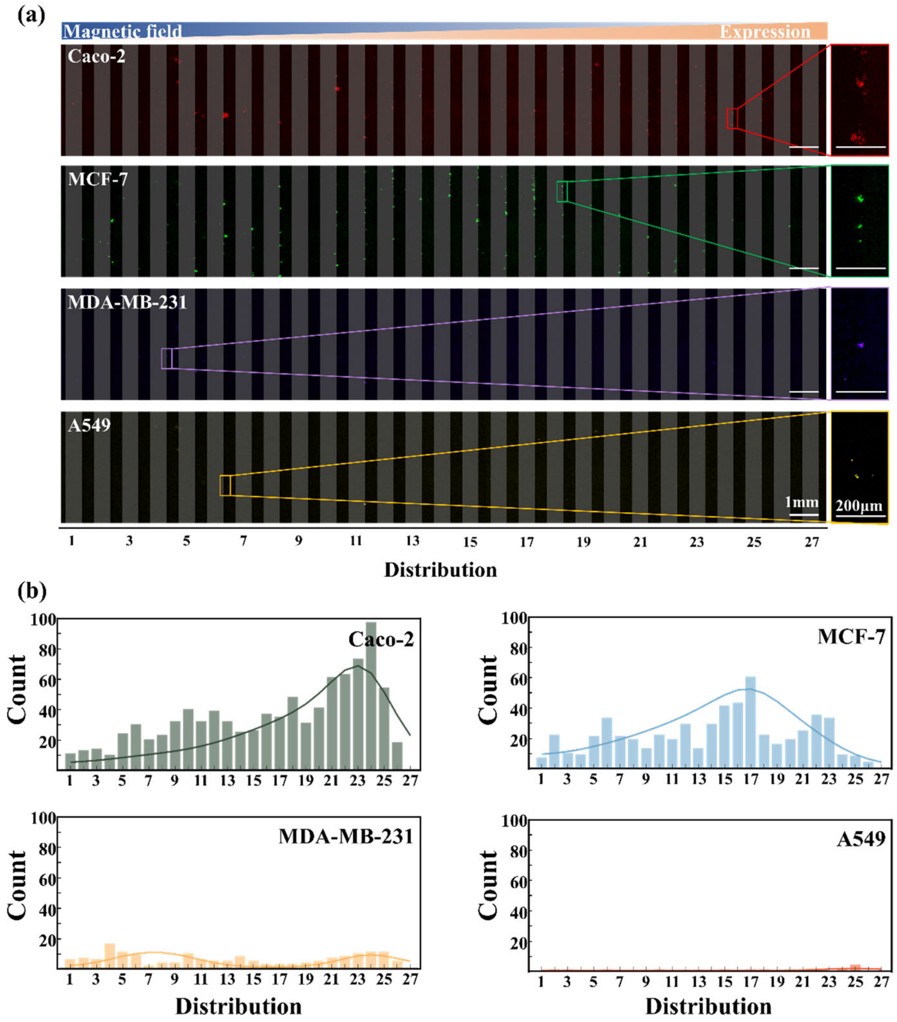

Figure S4. Stripe-resolved capture patterns and statistical distributions of EpCAM-defined subtypes for four epithelial cancer cell lines. (**a**) Representative fluorescence images of MagSculptor stripe-array capture patterns for Caco-2, MCF-7, MDA-MB-231 and A549 cells. For each cell line, approximately 1000 cells were processed in a single capture run ($n = 1$) and the positions of the captured cells along the 27-strip array were recorded. The imposed magnetic field forms a progressive gradient from stripe 1 (upstream, high-field region) to stripe 27 (downstream, low-field region), enabling graded capture according to EpCAM-associated bead loading. Scale bar for the full strip arrays: 1 mm; scale bar for inset views: 200 µm. (**b**) Stripe-wise histograms of captured cell count for the four cell lines shown in (a). The horizontal axis indicates the stripe index (1–27), and the vertical axis (Count) denotes the number of cells captured on each strip in the representative experiment. For interpretation, stripes 1–9, 10–18 and 19–27 correspond to the high-, medium- and low-field regions (H, M and L), respectively, such that increasing stripe index generally reflects progressively lower EpCAM labelling levels. These distributions are presented as descriptive statistics from a single representative run per cell line ($n = 1$) and are used to illustrate qualitative differences in capture position and expression-resolved heterogeneity across the four lines.

Cell line	MDA-MB-231	A549	Caco-2	MCF-7
Captured cells (number)	174	15	929	582
H region (stripes 1–9)	61 (35.1%)	8 (53.3%)	438 (47.1%)	149 (25.6%)
M region (stripes 10–18)	48 (27.6%)	4 (26.7%)	314 (33.8%)	278 (47.8%)
L region (stripes 19–27)	65 (37.4%)	3 (20.0%)	177 (19.1%)	155 (26.6%)
Mean stripe index ± SD	14.7 ± 8.2	10.8 ± 8.2	11.5 ± 6.8	14.0 ± 6.4
Median stripe index (IQR)	15.0 (17.0)	8.0 (12.0)	10.0 (12.0)	13.0 (10.0)

Table S1. Stripe-wise capture statistics for four epithelial cancer cell lines, each loaded at approximately 1000 cells per run in a single representative experiment (n = 1 independent capture run per line; the number of analyzed cells is given in the "Captured cells (number)" row). For each line, the table lists the total number of captured cells, the counts and percentages in the high-, medium-, and low-field regions (H: stripes 1–9; M: stripes 10–18; L: stripes 19–27), and summary stripe index statistics (mean ± s.d., median, and interquartile range, IQR) calculated over individual captured cells. **MDA-MB-231** displayed the broadest stripe-wise distribution, with captured cells spanning all three regions (H/M/L = 35.1%/27.6%/37.4%) and an interquartile range (IQR) of 17 stripe units (median = 15). This pattern is consistent with an overall low EpCAM labelling level but pronounced intra-line heterogeneity, i.e., the coexistence of rare highly labelled cells and a substantial fraction of weakly labelled cells within the same line. **Caco-2** cells were strongly enriched in the upstream high-field region (H/M/L = 47.1%/33.8%/19.1%; median stripe index = 10, IQR = 12), reflecting their high EpCAM expression. Kruskal–Wallis analysis followed by Dunn's multiple comparisons confirmed that the stripe index distribution of Caco-2 differed significantly from both MDA-MB-231 ($p = 2.4 \times 10^{-6}$) and MCF-7 ($p = 9.6 \times 10^{-13}$). **MCF-7** cells showed an intermediate pattern, with most captured cells residing in the mid-field region (H/M/L = 25.6%/47.8%/26.6%; median = 13, IQR = 10). This distribution was significantly downstream of Caco-2 ($p = 9.6 \times 10^{-13}$) but not statistically different from MDA-MB-231 in terms of the mean stripe index, suggesting a moderate EpCAM expression level with less extreme heterogeneity than MDA-MB-231. Only a small number of **A549** cells were captured across all stripes (N = 15), consistent with the known low EpCAM expression of this line. Within this limited dataset, captured A549 cells were distributed across all three regions (H/M/L = 53.3%/26.7%/20.0%; median = 8, IQR = 12) but no statistically significant pairwise differences were detected between A549 and the other lines using Dunn's post hoc tests (all $p > 0.05$). We therefore primarily treat A549 as a qualitative low-EpCAM comparator rather than a quantitatively ranked subtype in this analysis. Data are from a single representative experiment per cell line (n = 1); values are reported as descriptive statistics (e.g., counts, percentages, mean stripe index ± IQR) and are not intended as formal hypothesis tests.

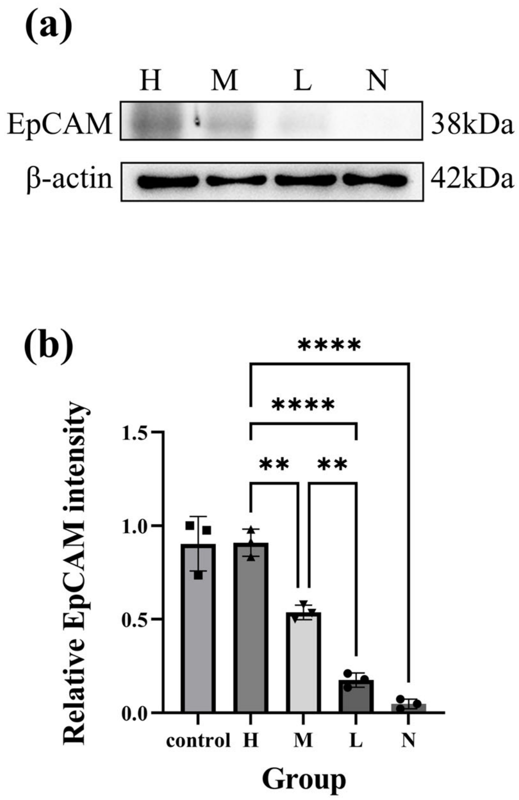

Figure S5. Western blot validation of EpCAM across MagSculptor-sorted MCF-7 subgroups. Data represent mean ± s.d. of three independent Western blots (n = 3). EpCAM band intensities were normalized to β-actin and then to the control group (unsorted total, set to 1.0). Ordinary one-way ANOVA with Tukey's post hoc test was used; only comparisons with $p < 0.05$ are indicated by asterisks (* $p < 0.05$, ** $p < 0.01$, *** $p < 0.001$, **** $p < 0.0001$).

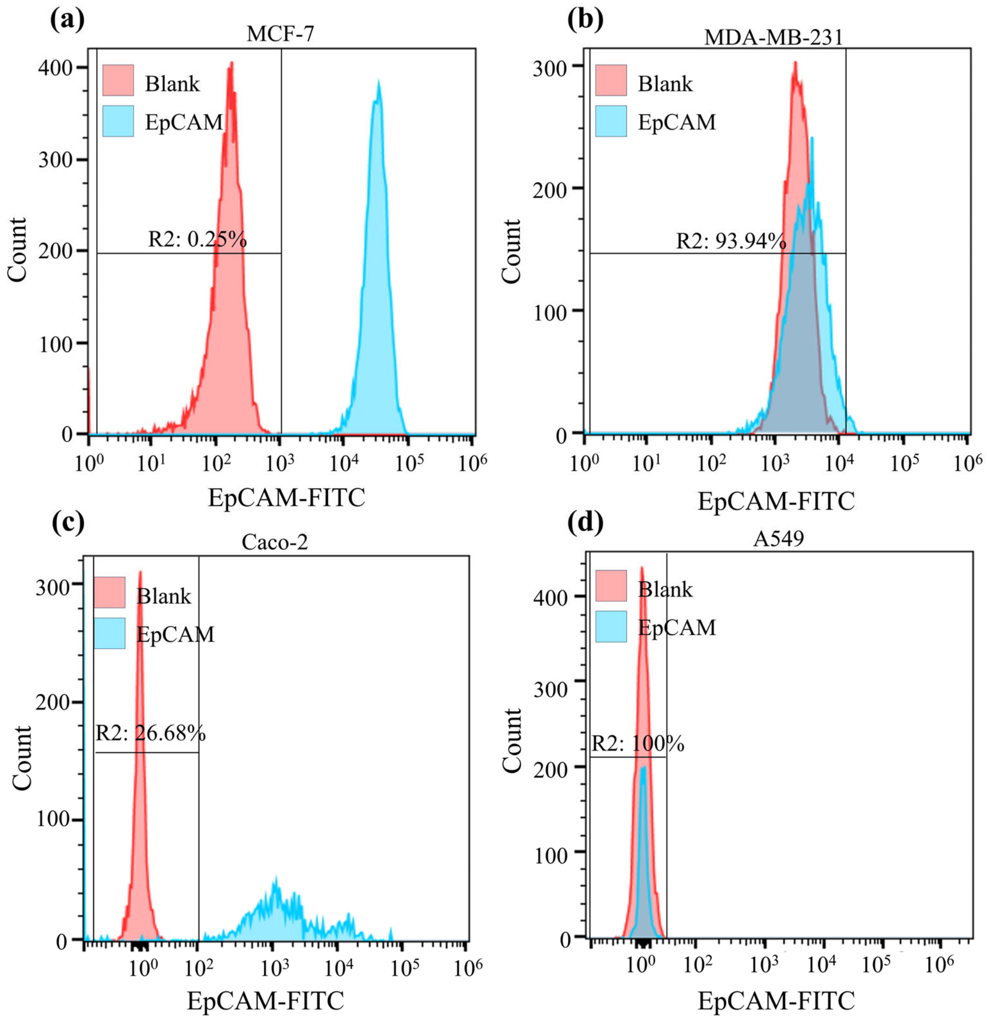

Figure S6. Flow cytometric assessment of EpCAM expression in MCF-7, MDA-MB-231, Caco-2 and A549 cell lines. Due to the characteristically low expression levels of EpCAM in the MDA-MB-231 and A549 cell lines, 93.94–100% of the cellular population exhibited fluorescence intensities that precluded definitive determination of fluorescent antibody contribution, rendering negative/positive discrimination indeterminate and compromising the reliability of flow cytometric analysis for this low-expressing phenotype.

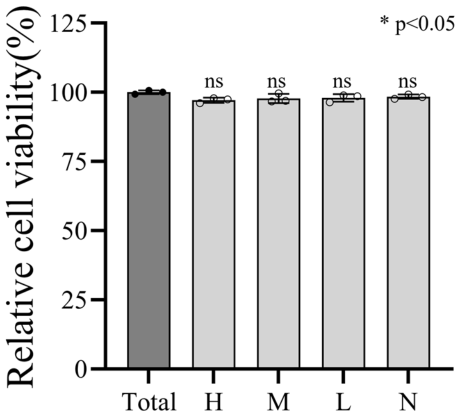

Figure S7. Assessment of cell viability following chip-based sorting operations ($n = 3$). Error bars represent mean ± s.d. * $p < 0.05$ versus 0% viability control; ns, not significant. Viability statistics were determined for pre-sorting samples across three independent separation experiments with corresponding post-sorting relative viability assessments conducted for each cellular subpopulation. Statistical analysis was conducted using a two-tailed Student's t-test in which the viability of each subgroup (H, M, L, and N) was compared against the total pre-sorting population. No statistically significant differences were observed between pre-sorting and post-sorting groups ($p > 0.05$), indicating that the microfluidic sorting process exerts negligible impact on overall cell viability.

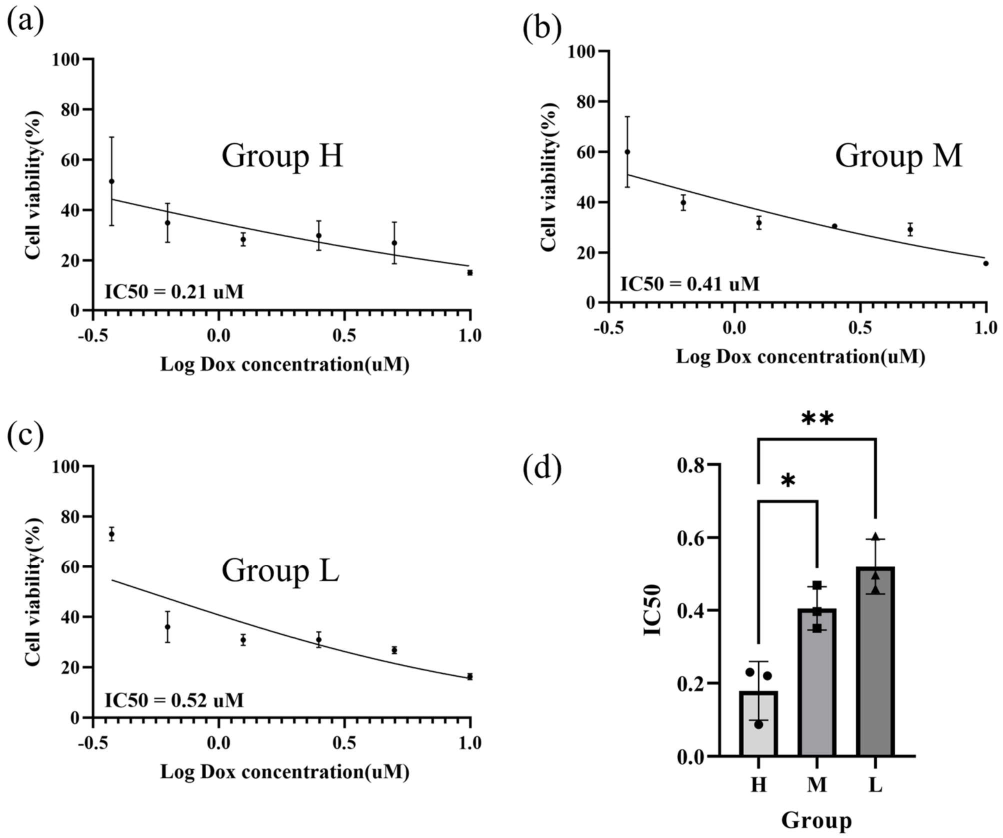

Figure S8. Doxorubicin response of MagSculptor-sorted MCF-7 subtypes. (**a–c**) Representative dose–response curves of high- (H), medium- (M), and low- (L) EpCAM-expressing MCF-7 subgroups after 48 h exposure to doxorubicin (0–10 μM). Cell viability was quantified by a CCK-8 assay and normalized to the 0 μM untreated control. Each data point represents the mean of three technical replicates from a single independent experiment and is shown here to illustrate the characteristic right shift of the curves from H to M to L. (**d**) Summary of half-maximal inhibitory concentration (IC_{50}) values for the three subgroups across independent experiments. Bars show mean ± s.d. (n = 3 independent experiments). For each experiment, IC_{50} values were obtained by nonlinear least-squares fitting of sigmoidal dose–response curves in GraphPad Prism. Statistical comparisons of IC_{50} across subgroups were performed using ordinary one-way ANOVA with Tukey's multiple comparisons test; only pairwise comparisons with $p < 0.05$ are indicated (* $p < 0.05$, ** $p < 0.01$). H, M, and L denote the high-, medium-, and low-EpCAM subpopulations sorted by MagSculptor.

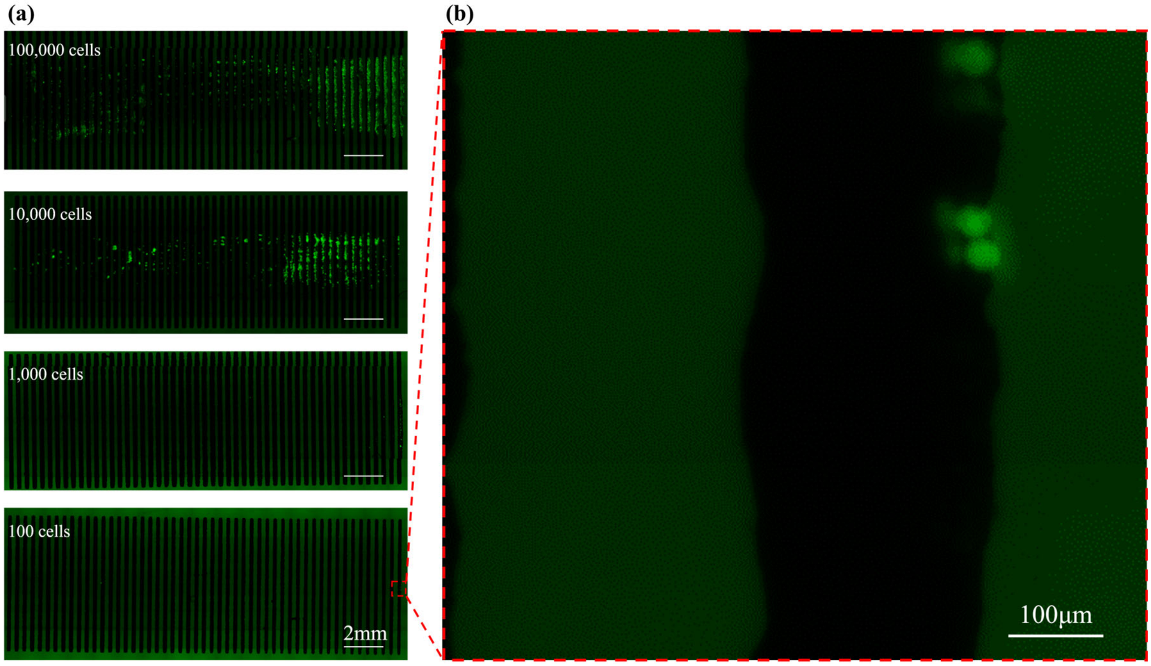

Figure S9. Capture of MDA-MB-231 cells from whole blood samples. (**a**) Capture profiles of EpCAM-positive cells following the introduction of varying quantities of MDA-MB-231 cells into whole blood with cellular enrichment based on EpCAM expression levels serving as the selection criterion. (**b**) High-magnification view of the sparse cell capture regions depicted in image (a), revealing individual cellular capture events at low target cell concentrations.

Ref. No.	Target (TIL/CTC/cell line)	Sorting dimensionality (binary/multi-level by antigen expression)	Typical processing throughput	Need complex microstructures? (micropillars, ratchets, etc.)	One-to-one mapping between expression level and spatial position?
**41**	Immunomagnetically labeled tumor cell lines and leukocytes	**Multi-level by antigen expression** (≈8 discrete magnetophoretic bins)	>5×10^2 cells·min^{-1} (single-cell profiling mode)	**No**—straight channel with only support pillars (not used as separation obstacles)	**Yes**—bin index monotonically reflects magnetophoretic mobility and membrane antigen density
**26**	Rare immune/tumor cells labeled with different antibody–magnetic bead cocktails	**Multi-level/multimodal profiling by binding capacity** (continuous spectrum of magnetic content)	~ 3×10^4 cells·min^{-1} with ~96% recovery and ~99% purity	**No**—rectangular microchannel, no internal obstacles	**Yes**—lateral displacement and collection position correlate quantitatively with antigen-binding/magnetic content
**42**	CTCs in whole blood (label-free, antigen-independent)	**Binary** (CTC-enriched vs. background leukocytes; based on size and magnetization)	~ 10 to 12 mL·h^{-1} whole blood (clinical-scale enrichment)	**No**—straight channel between external magnets	**No**—separation based on biophysical properties, not on graded antigen expression
**43**	Model cancer cells vs. blood cells (label-free)	**Binary/few-level physical separation** (size, deformability, magnetization)	Up to ~ 10^5 cells·s^{-1}; tens of mL·h^{-1} sample volume	**No**—simple rectangular channel; no intricate microstructures	**No**—does not resolve surface expression levels; focuses on label-free physical contrast
**44**	EpCAM$^+$ CTCs from whole blood	**Binary** (captured vs. uncaptured CTCs by affinity)	~ 10 mL·h^{-1} whole blood	**Yes**—radial flow geometry and structured capture region	**No**—affinity capture above an effective threshold; no explicit mapping of expression level to position
**45**	Immunomagnetically labeled rare mammalian cells (model rare-cell mixtures)	**Binary/few-stream enrichment** (different magnetophoretic mobilities)	μL–mL·h^{-1} regime (rare-cell demonstration)	**No**—straight channel with external permanent magnet	**No**—although magnetophoretic mobility is continuous, the device is not used as a quantitative expression–position profiler
**46**	Immunomagnetically labeled cells (e.g., leukocyte subpopulations)	**Binary / stepwise enrichment** (target vs. non-target)	μL–mL·h^{-1} flows (rare-cell separation)	**Yes**—patterned permanent micromagnets integrated near the microchannel	**No**—designed for efficient capture/release rather than graded expression mapping
**47**	Magnetic vs. non-magnetic cells and beads	**Binary/limited multi-stream** (magnetic vs. non-magnetic plus few intermediate trajectories)	μL·min^{-1} continuous free-flow operation	**No**—open free-flow chamber without micro-pillars or ratchets	**No**—magnetization level affects deflection, but the system is not calibrated as an antigen-expression profiler
**48**	EpCAM-labeled CTCs from blood	**Multi-level based on antigen expression** (discrete EpCAM-high/intermediate/low sub-populations)	mL·h^{-1}-scale processing of clinically relevant blood volumes	**Yes**—two-stage structure (magnetophoretic pre-separation + spatially resolved capture regions)	**Yes**—CTCs are partitioned into discrete outlets corresponding to different EpCAM expression/magnetic labeling levels
**49**	CTCs spiked into blood with **low/medium/high** antibody expression	**Multi-level based on antigen expression** (three discrete expression bins)	μL–mL·h^{-1} flow rates (laminar microfluidic regime)	**Yes**—divergent serpentine channel geometry with position-dependent magnet distance	**Yes**—three outlets correspond to low, intermediate, and high expression levels (quasi-continuous profiling with three bins)
**50**	CD4$^+$ T cells, monocytes and neutrophils (cell therapy-relevant leukocyte populations)	**Multi-level based on antigen density** (CD4-high vs. CD4-low and intermediate)	10^8–10^9 target cells·h^{-1} (throughput 3–10× faster than FACS)	**Yes**—disposable cartridges with dense arrays of ferromagnetic microstructures (‘ratcheting cytometry’)	**Yes**—cells are fractionated into a series of “digital” magnetic bins whose index reflects bound magnetic content and hence antigen density

Table S2. Representative magnetophoretic, ferrohydrodynamic and immunomagnetic microfluidic platforms for rare cell separation and phenotypic/antigen-level profiling. For each work, the table summarizes the target cell type (e.g., CTCs, leukocyte subpopulations, model cell lines), the sorting dimensionality (binary enrichment of “positive vs. negative” cells versus multi-level fractionation based on antigen expression), the typical processing throughput reported by the authors, whether complex internal microstructures such as dense micropillar or ratchet arrays are required, and whether a monotonic one-to-one mapping between antigen expression (or magnetic loading) and spatial position is explicitly implemented.

Most earlier devices with very high volumetric throughput [42–44] operate essentially in a binary mode (enriching a single “target” fraction), while several pillar-free magnetophoretic systems [45–47] achieve continuous magnetophoretic deflection but are not calibrated as quantitative expression–position profilers. Only a subset of recent platforms [41,26,48–50] explicitly resolves

multiple subpopulations according to antigen density, and these often rely on relatively complex architectures (graduated capture regions, divergent serpentine channels, or dense ferromagnetic micro-arrays) to encode expression levels. In contrast, the present MagSculptor device combines (i) multi-level subtyping within a single cell line based on EpCAM expression, (ii) a clinically relevant sample throughput on the order of 4 $mL \cdot h^{-1}$ (~10^4 $cells \cdot min^{-1}$), and (iii) a structurally simple, pillar-free microchannel in which a sculpted lateral magnetic field provides a calibrated mapping from magnetophoretic displacement to immunomagnetic labeling level.

Abbreviations: TIL, tumor-infiltrating lymphocyte; CTC, circulating tumor cell; FACS, fluorescence-activated cell sorting.
